# Label-free imaging and biomarker analysis of exosomes with plasmonic scattering microscopy†

**DOI:** 10.1039/d2sc05191e

**Published:** 2022-10-12

**Authors:** Pengfei Zhang, Jiapei Jiang, Xinyu Zhou, Jayeeta Kolay, Rui Wang, Zijian Wan, Shaopeng Wang

**Affiliations:** Biodesign Center for Bioelectronics and Biosensors, Arizona State University Tempe Arizona 85287 USA Shaopeng.Wang@asu.edu; Beijing National Laboratory for Molecular Sciences, Key Laboratory of Analytical Chemistry for Living Biosystems, Institute of Chemistry, Chinese Academy of Sciences Beijing, 100190 China; School of Biological and Health Systems Engineering, Arizona State University Tempe Arizona 85287 USA; State Key Laboratory of Bioelectronics, School of Biological Science and Medical Engineering, Southeast University 2 Sipailou Nanjing 210096 China; School of Electrical, Energy and Computer Engineering, Arizona State University Tempe Arizona 85287 USA

## Abstract

Exosome analysis is a promising tool for clinical and biological research applications. However, detection and biomarker quantification of exosomes is technically challenging because they are small and highly heterogeneous. Here, we report an optical approach for imaging exosomes and quantifying their protein markers without labels using plasmonic scattering microscopy (PSM). PSM can provide improved spatial resolution and distortion-free image compared to conventional surface plasmon resonance (SPR) microscopy, with the signal-to-noise ratio similar to objective coupled surface plasmon resonance (SPR) microscopy, and millimeter-scale field of view as a prism-coupled SPR system, thus allowing exosome size distribution analysis with high throughput. In addition, PSM retains the high specificity and surface sensitivity of the SPR sensors and thus allows selection of exosomes from extracellular vesicles with antibody-modified sensor surfaces and *in situ* analyzing binding kinetics between antibody and the surface protein biomarkers on the captured exosomes. Finally, the PSM can be easily constructed on a popular prism-coupled SPR system with commercially available components. Thus, it may provide an economical and powerful tool for clinical exosome analysis and exploration of fundamental issues such as exosome biomarker binding properties.

## Abstract 2

The scattering imaging scheme permits the conventional prism-based SPR system to analyse single extracellular vesicles and determine the molecular profiles to characterize the exosomes secreted from different cells.

## Graphical abstract

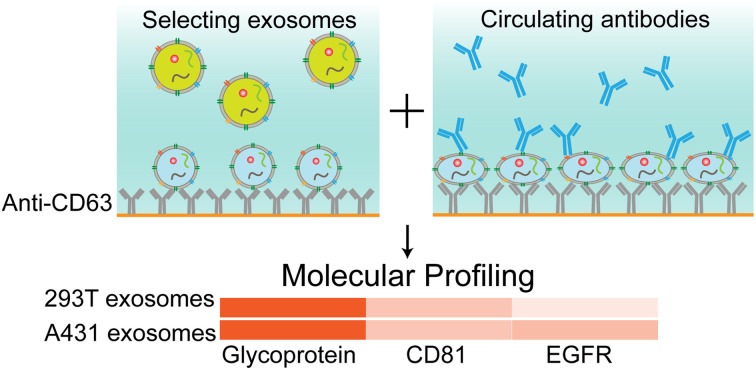

## Introduction

All prokaryotic and eukaryotic cells shed massive quantities of extracellular vesicles (EVs) into circulation. The exosome is one particular type of EV with a diameter range of 30 to 150 nm (average ∼ 100 nm) and serves as an intercellular transit system to regulate distant cell physiology and activity.^1^ Due to the endosomal origin mechanism, the constituents of exosomes can reflect the type of cells where they are released. Thus, rapid exosome analysis provides an effortless and promising way to evaluate the health conditions without biopsying the tissues.^1–10^

Traditional exosome biomarker analysis relies on the western blot technique, which requires extensive post-labelling processes for detection, making it impractical for rapid detection. Besides, western blot detection cannot analyse the size information of the exosomes, which is usually achieved with an additional nanoparticle tracking analysis measurement. Several approaches have been developed in the recent decade for rapid exosome biomarker analysis, such as miniaturized nuclear magnetic resonance,^11,12^ surface plasmon resonance (SPR) array sensors,^13^ nanoplasmonic enhanced scattering,^14^ target magnification with reagent-loaded liposomes,^15^ electrochemical nanosensors,^16^ molecular rotors,^17^ upconversion nanoparticles,^18^ fluorescence imaging,^19^ interferometric reflectance imaging sensors,^20^ prism-coupled SPR imaging sensors,^21,22^ and SPR microscopy.^23,24^ Among these technologies, SPR shows promising potential for multiplexed rapid exosome analysis to determine multiple biomarkers and exosome size distribution in one system. First, the SPR has a probing depth of ∼100 nm, which is matched to exosome size for improved detection sensitivity. The content level and binding kinetics of multiple biomarkers can be quantified by real-time monitoring of the SPR sensor response to different antibody solutions flowing onto the exosomes immobilized on the sensor surface.^22,25,26^ Second, the microscopic SPR imaging system, namely, the SPR microscopy, can provide exosome size information by tracking the image intensity of single exosomes. However, there are still some limitations to the wider application of SPR approaches. First, the prism-coupled SPR imaging sensor can easily provide the millimetre-scale field of view to capture a large number of exosomes for multiplexed biomarker analysis,^21,22,27,28^ but it cannot provide enough spatial resolution or signal-to-noise ratio (SNR) for exosome size determination.^23^ The SPR microscopy constructed from an oil immersion objective with a high numerical aperture (NA) allows the single exosome analysis, but the high NA objective usually has a large magnification, resulting in a small field of view and, therefore, the limited throughput.^29–32^ Second, the surface plasmonic waves have long decaying length along the surface, and thus, the SPR microscopy has a parabolic tail-shaped point spread function, resulting in low spatial resolution, and making it challenging to process the images automatically with regular image processing tool.^33–35^

Herein, we report plasmonic scattering microscopy (PSM) technology for imaging exosomes, analysing multiple protein biomarkers, and quantifying their binding kinetics without labels. PSM was first developed to realize label-free single-molecule imaging on SPR microscopy, where it has been demonstrated that PSM can provide Gaussian distributed point spread function for high spatial resolution and automatic image processing with conventional software such as ImageJ.^36–42^ In this article, we demonstrated that the PSM constructed on the popular prism coupled SPR system can provide an SNR similar to SPR microscopy, millimetre-scale field of view as a prism-coupled SPR system, and Gaussian distributed point spread function, thus allowing exosome size distribution analysis automatically with a conventional open-source tool and capturing a large number of exosomes for biomarker analysis. PSM also retains the high specificity and surface sensitivity of the SPR sensors and thus allows selecting exosomes from extracellular vesicles with antibody-modified sensor surfaces and *in situ* analysing binding kinetics between antibody and the surface protein biomarkers on the captured exosomes.

## Results and discussion

### Imaging principle

**Fig. 1:**
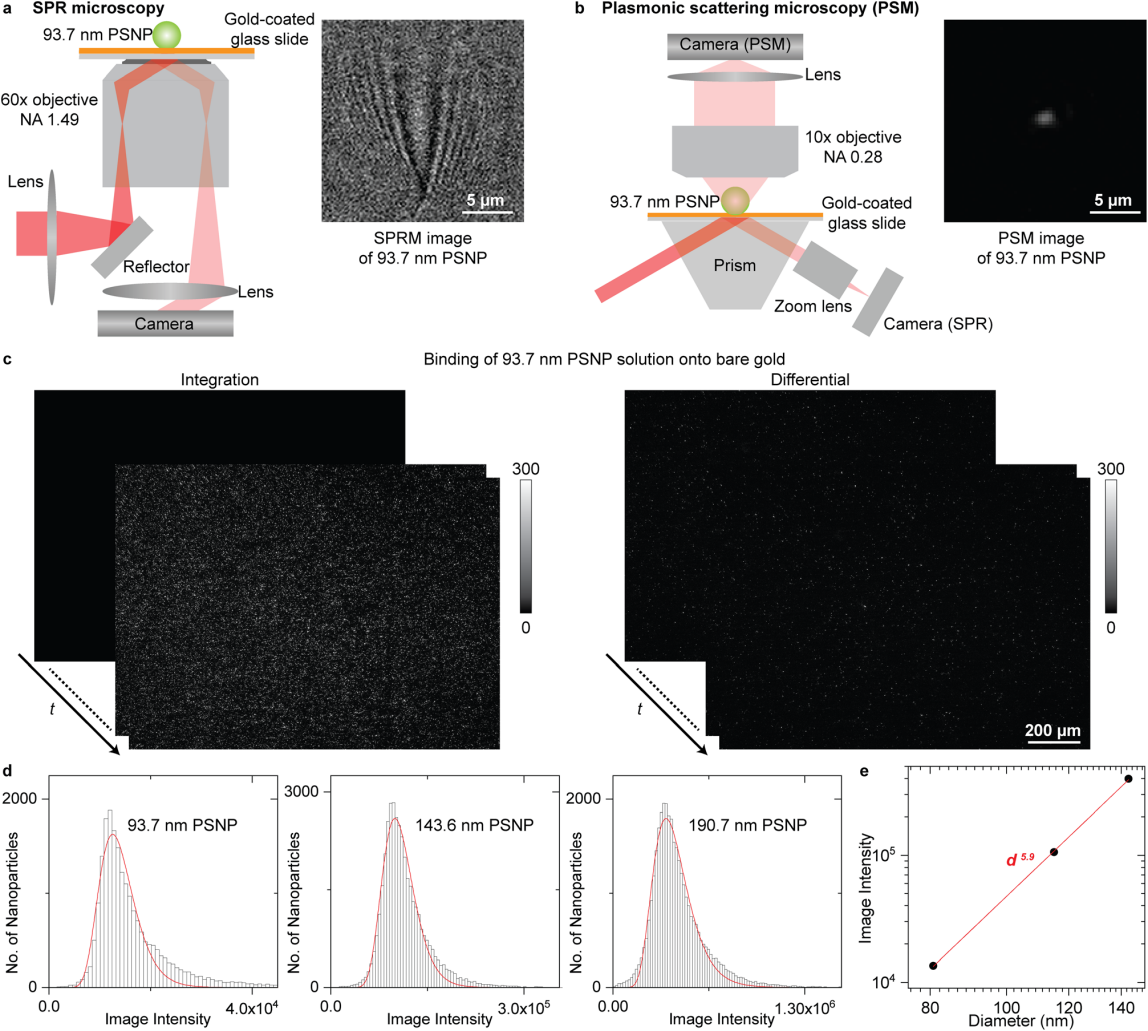
(a) and (b) Simplified sketches of the optical setups for SPR microscopy (a) and PSM (b), and their images of one 93.7 nm polystyrene nanoparticle (PSNP). (c) Integrated and differential PSM images monitoring the binding of 93.7 nm PSNP onto the gold surface. The dynamic process is shown in Movie S1.† (d) PSM image intensity histograms of the PSNP with different diameters, where the solid lines are lognormal fittings and the mean image intensities are marked. (e) Mean PSM image intensity *versus* PSNP diameter. The *z*-distance dependence of surface plasmonic waves is considered (Note S1†). Incident light intensity is 0.1 W cm^−2^ for SPR microscopy and 4 W cm^−2^ for PSM.

The SPR microscopy employs oblique illumination to excite the surface plasmonic wave on the gold-coated glass slide with a 60× oil-immersion objective (Fig. 1a), where the reflected light is recorded to form an SPR microscopic image. The image contrast is determined by the interference between the surface plasmonic wave and the plasmonic wave scattered by the analyte.^36^ The surface plasmonic wave has ∼10 μm propagation length with the incident wavelength of 660 nm, leading to a parabolic tail following the spot at the location of the analyte in the SPR image, which is hard to be processed with regular software and can only be automatically analysed with specifically designed image processing algorithms.^23,34,35^ The PSM is constructed differently from SPR microscopy by employing one objective to observe the plasmonic wave scattered by analytes on the top of the gold-coated glass slide (Fig. 1b and S1† for details). The reflection beam was also recorded simultaneously to create SPR sensor output. The PSM does not record the surface plasmonic waves. Thus, its point spread function is Gaussian distributed as classical optical microscopy, providing higher spatial resolution than SPR microscopy even with a low aperture numerical dry objective (Fig. S2†) and making it easy to perform the image processing using conventional software such as ImageJ. In addition, the PSM can employ the prism configuration for a large illumination area and a low magnification objective, such as the 10× dry objective in this study, to achieve a large field of view. Considering that the incident light will occupy half view of the objective used in SPR microscopy, the PSM with a 10× dry objective will provide ∼40 times larger field of view than SPR microscopy with the same imaging path, making it easy to capture a large number of exosomes for biomarker analysis as the conventional prism coupled SPR sensor.^21,22^

One key advantage of SPR technology is the high measurement sensitivity, which is usually defined by the SNR for imaging single nanoparticles and refractive index resolution for ensemble binding kinetics analysis.^43,44^Fig. 1c presents the integrated and differential PSM images monitoring the binding of 93.7 nm polystyrene nanoparticles onto the gold surface, where the dynamic process is shown in Movie S1.† After flowing the polystyrene nanoparticles dispersed in the PBS buffer into the channel, the nanoparticles will bind onto the bare gold surface automatically, which may be due to that the charge screening effect in PBS buffer greatly reduces the electrostatic repulsion force between negatively charged PS particles and the double-layer water on the gold surface that prefer to orient with the negatively charged oxygen atom toward the solution.^45^ The differential images were achieved by subtracting each image with previous frame. The image intensities of single-binding events on the differential frames were tracked with TrackMate plugin in ImageJ for building the intensity histogram to suppress the interference from two nanoparticles binding to the nearby surface with distance smaller than the diffraction limit. The PSM image intensity of each binding event was determined by integrating the intensities of all pixels covered by the bright spot created by the analyte. To further estimate the imaging SNR, the PSM system was calibrated by imaging polystyrene nanoparticles with different diameters (Fig. 1d). The hydrodynamic diameters of nanoparticles were confirmed by dynamic light scattering to ensure no aggregations in the sample. Taking the *z*-distance dependence of surface plasmonic wave into consideration (Note S1†), the PSM image intensity scales with *d*^5.9^, where *d* is the diameter, and the exponent is close to six (Fig. 1e). This is expected because the light scattering dominates the PSM image contrast. Then, dividing the image intensity by the background fluctuation, the SNR of PSM measurement for imaging 93.7 nm polystyrene nanoparticles can be determined to be ∼145 (Note S2†), which is comparable to the state-of-art SPR microscopy,^23^ indicating that it can provide sufficient SNR for analyzing single exosome size. To estimate the refractive index resolution for ensemble measurements, the solutions with different refractive indices were measured serially, and the refractive index resolution of PSM and SPR channel in this system can be estimated to be ∼4.3 × 10^−6^ RIU, and ∼6.4 × 10^−6^ RIU, respectively (Fig. S3†), which is comparable to most ensemble SPR sensors.^44,46,47^ The RIU represents the refractive index unit.

### Measurement of exosome binding to anti-CD63

**Fig. 2:**
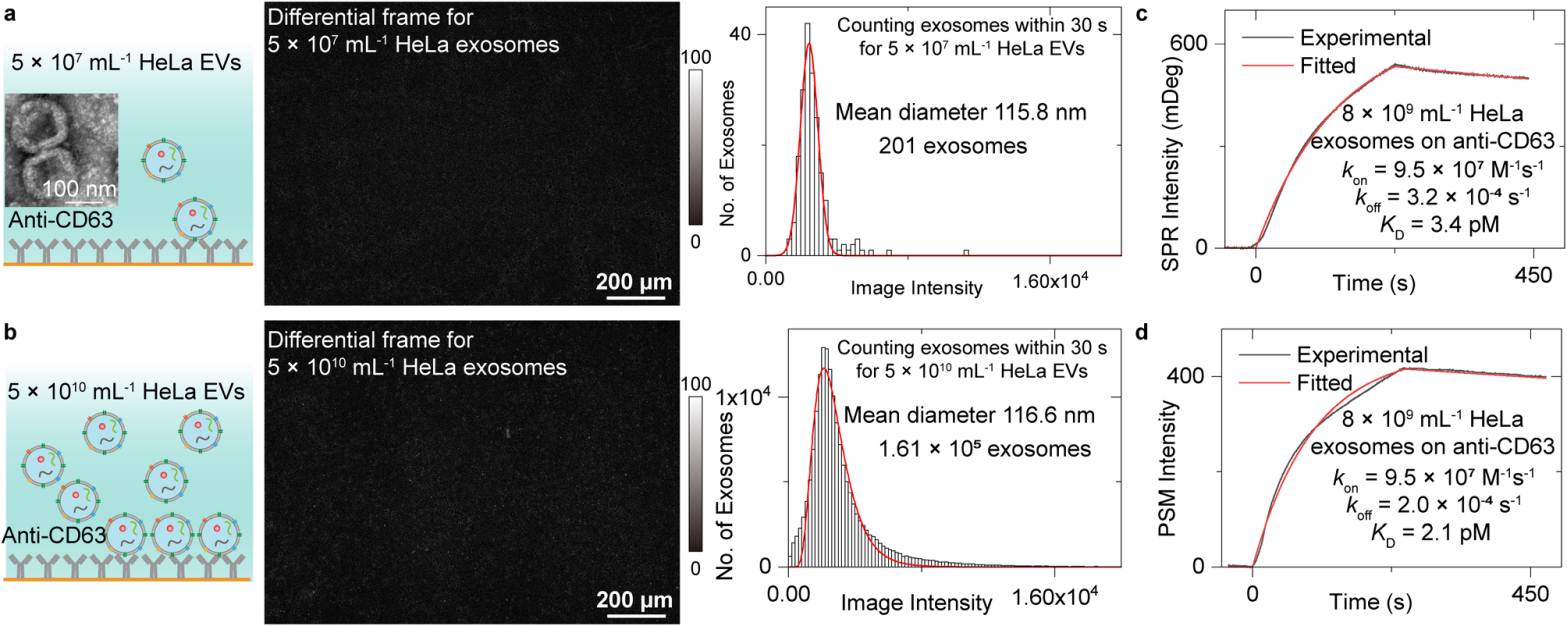
(a) and (b) TEM images of EVs from HeLa cells, schematic of 5 × 10^7^ mL^−1^ (a) and 5 × 10^10^ mL^−1^ (b) exosomes binding to anti-CD63-modified surface, differential frames, and PSM image intensity histograms of the exosomes by individually counting the single-binding events within first 30 seconds. The mean diameter of exosomes and sample size are also presented. The solid lines in histograms are Gaussian (a) or lognormal (b) fittings. (c) and (d) Ensemble SPR (c) and PSM (d) measurement of 5 × 10^10^ mL^−1^ HeLa EVs binding to the anti-CD63 antibody, where the exosome concentration of 8 × 10^9^ mL^−1^ determined from binding frequency is used for fitting.

We first analysed the exosome size distribution and binding kinetics by flowing two EV solutions with the concentration of 5 × 10^7^ mL^−1^ and 5 × 10^10^ mL^−1^ onto the anti-CD63 antibody-modified PSM sensor surface (Fig. 2a and b). The EVs were extracted from the media culturing the HeLa cells by ultracentrifuge and resuspended in the PBS buffer (Methods). CD63 is a commonly used exosome surface protein marker, and thus, anti-CD63 can recognize the exosomes from other kinds of EVs. One differential frame recording 5 × 10^7^ mL^−1^ EVs binding to the surface is shown in Fig. 2a, where only 1 or 2 binding events are observed. Another differential frame recording 5 × 10^10^ mL^−1^ EVs binding to surface is shown in Fig. 2b, where over 100 binding events can be observed. To avoid the effect of resonance angle shift resulting from exosome binding, only the binding events within the first 30 seconds were analysed to build the intensity histograms, where the mean intensity was achieved by Gaussian or lognormal fitting. After considering the *z*-distance dependence of surface plasmonic wave (Note S1†) and the refractive index difference between polystyrene nanoparticles and EV (Note S3†),^48^ the mean diameter of the exosomes can be estimated to be ∼115.8 nm for 5 × 10^7^ mL^−1^ EV solution and ∼116.6 nm for 5 × 10^10^ mL^−1^ EV solution using the calibration curve shown in Fig. 1e. This value agrees with the exosome size estimated by NTA analysis (Fig. S4†) and previously reported values,^1^ demonstrating the exosome size analysis capability of the PSM. From the total binding events, the exosome concentration can be estimated to be ∼8 × 10^9^ mL^−1^ for 5 × 10^10^ mL^−1^ EV solution and ∼1 × 10^7^ mL^−1^ for 5 × 10^7^ mL^−1^ EV solution based on Fick's law of diffusion (Note S4†).^49^ The exosome concentration is notably lower than the total concentration of extracellular vesicles. This is reasonable because there exist other kinds of vesicles, such as ectosomes, apoptotic bodies, and microvesicles.^1,3,24^

Then, the binding kinetics of exosome binding to anti-CD63 can be analysed by simultaneously monitoring the ensemble SPR and PSM image intensity along with flowing the 5 × 10^10^ mL^−1^ HeLa EV solution onto the anti-CD63 antibody-modified sensor surface and then flowing PBS buffer over the sensor surface to allow study of dissociation of the exosomes from anti-CD63. The SPR and PSM image intensity variations were recorded in real time to produce a binding kinetics curve. Fitting of the curves with the first-order binding kinetics model determines the association (*k*_on_), dissociation (*k*_off_) rate constants, and the equilibrium dissociation constant (*K*_D_ = *k*_off_/*k*_on_). The exosome concentration of 8 × 10^9^ mL^−1^ determined from binding frequency is used for fitting. These values achieved by PSM are similar to ensemble SPR, demonstrating that the PSM can also provide high sensitivity for ensemble measurements. The *K*_D_ is very small, indicating that the exosomes can bind to anti-CD63 tightly, which is likely due to the multivalency bindings from multiple CD63 binding sites per exosome.^13^

Due to the random orientation of antibody, the functional anti-CD63 coverage is ∼50% in Fig. 2, where high antibody coverage was employed to ensure sufficient capture probability. Considering that the analyte is usually larger than the antibody, this coverage has been demonstrated to efficiently block the nonspecific binding.^36,37^ To demonstrate this, we flow the EV solution onto the goat anti-mouse IgG-modified surface, and very few binding events were observed (Fig. S5†).

The exosomes bind to the sensor surface *via* multiple binding sites, so the binding kinetics differ with antibody coverage. As one demonstration, we can see that no obvious dissociation was observed within the measurement period after exposing the sensor surface to 5 × 10^7^ mL^−1^ EV solution, where each exosome should bind to the surface *via* more binding sites than the experiment measuring high concentration EV solution (Fig. S6†). In addition, if we mix BSA into the anti-CD63 solution when modifying the sensor surface to reduce antibody coverage, the binding affinity of exosomes was measured to be notably lower compared with the values achieved on sensor surface with high antibody coverage (Fig. S7†).

### Measurement of WGA binding to exosomes

**Fig. 3:**
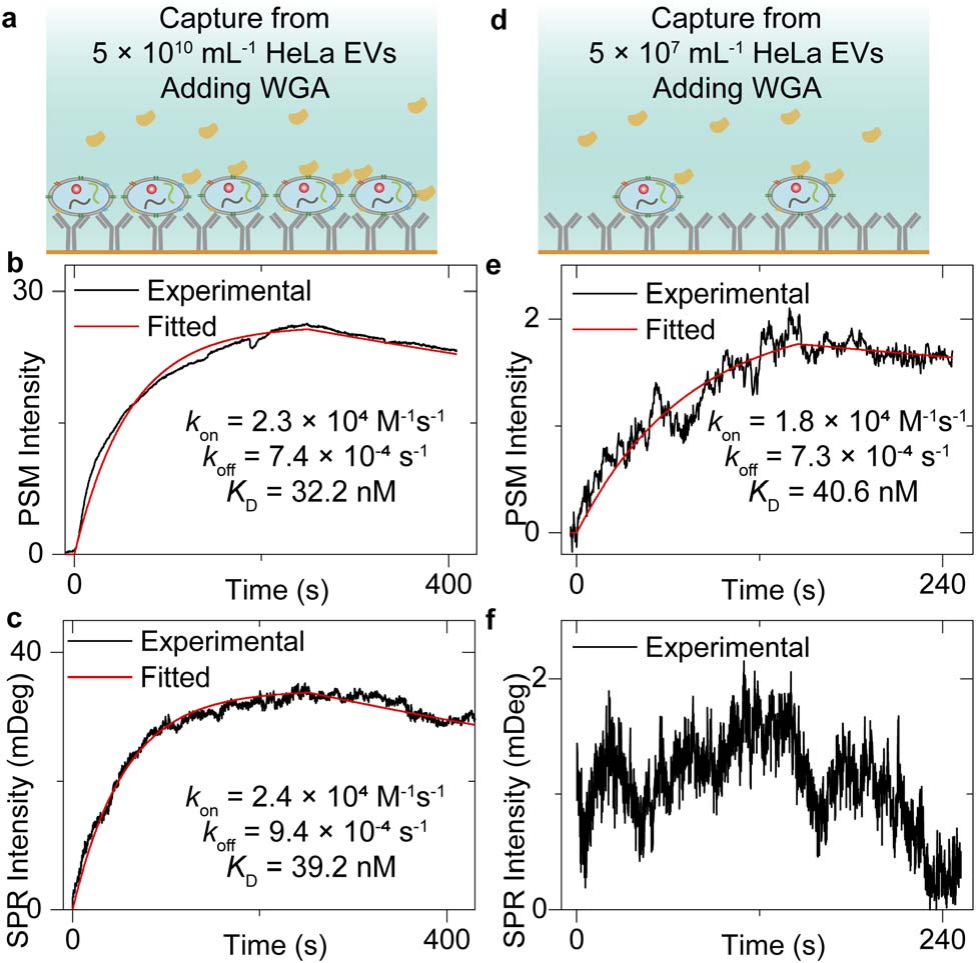
(a) and (d) Schematic of WGA binding to exosomes captured from 5 × 10^10^ mL^−1^ (a) and 5 × 10^7^ mL^−1^ (d) EV solution. (b) and (e) Ensemble PSM measurement of WGA binding onto the *N*-acetylglucosamine and sialic acid groups on the surfaces of HeLa exosomes captured from 5 × 10^10^ mL^−1^ (b) and 5 × 10^7^ mL^−1^ (e) EV solution. (c) and (f) Ensemble SPR measurement of WGA binding onto the *N*-acetylglucosamine and sialic acid groups on the surfaces of HeLa exosomes captured from 5 × 10^10^ mL^−1^ (c) and 5 × 10^7^ mL^−1^ (f) EV solution.

Fig. 2 has shown that the exosomes can bind to the surface tightly, which indicates that the sensor surface absorbing the exosomes can also provide stable background intensity, thus allowing the study of the molecular interactions of the membrane proteins on the exosome surface. To demonstrate this, after flowing the HeLa EV solution onto the anti-CD63-modified sensor surface and counting single-binding events at the first 30 seconds to achieve the image intensity histogram (Fig. 2), the PBS buffer was flowed onto the sensor surface for 20 minutes to allow the vesicle deformations.^50^ Next, the incident angle was adjusted to the position allowing the maximum PSM intensity response to refractive index variations (working point shown in Fig. S2†), and the 25 μg mL^−1^ wheat germ agglutinin (WGA) in PBS buffer was flowed onto the exosomes immobilized on the sensor surface to observe the WGA binding to *N*-acetylglucosamine and sialic acid groups of glycoproteins on the exosome surfaces (Fig. 3a and d). Finally, the PBS buffer was flowed onto the exosomes to allow the dissociation of WGA from surface targets. The exosome response to the WGA association and dissociation was recorded in real time simultaneously by SPR and PSM to produce a binding kinetics curve (Fig. 3b, c, e, and f). Due to the mechanical drift, pump noise, and shot noise, there are some fluctuations in the binding curves. For the SPR measurement of WGA binding to the exosomes captured from low concentration EV solution, the fluctuations will block the signals. Meanwhile, the PSM presents higher SNR than SPR because the PSM signal contains analyte scattering signals in addition to the SPR condition variation signals, which produces the ensemble SPR sensor output.^41^ In addition, the PSM can also recognize the exosome binding sites owing to high spatial resolution and measure the signals resulting from WGA binding onto these areas to suppress the interference from the blank regions. Fitting of the curves with the first-order binding kinetics model determines the *k*_on_, *k*_off_, and *K*_D_. These values are similar for both SPR and PSM measurements, and in good agreement with the previously reported results measuring the WGA binding to membrane proteins,^32,51,52^ indicating that the PSM can measure the binding of proteins to the markers on the surface of exosomes immobilized on the sensor surface.

### Biomarker profiling

**Fig. 4:**
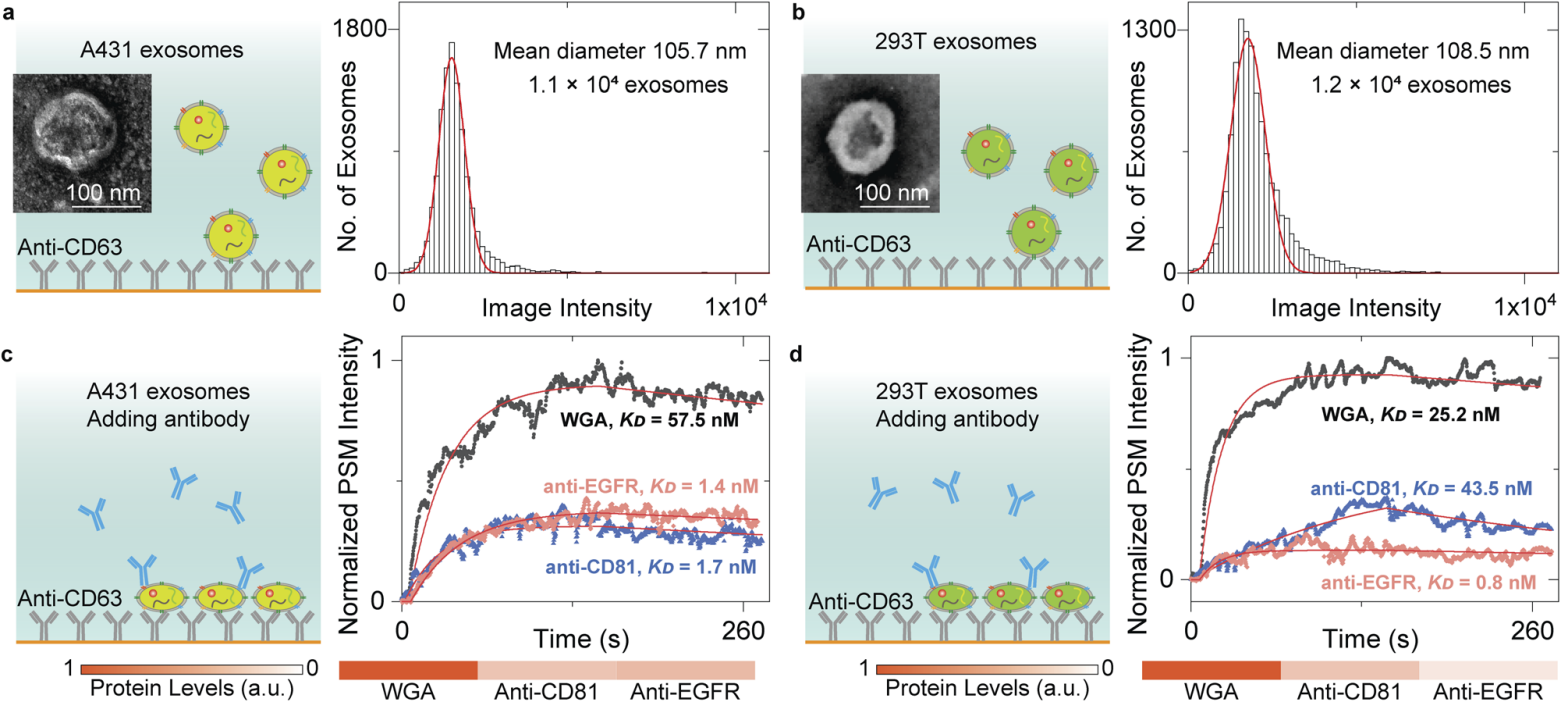
(a) and (b) TEM images of EV from A431(a), and 293T(b) cells and PSM image intensity histograms of the exosomes by individually counting the single-binding events. The exosomes are recognized by the anti-CD63 antibody immobilized on the gold surface. The mean diameter of exosomes and sample size are presented in the figure. (c) and (d) Ensemble PSM measurements of WGA, anti-CD81, and anti-EGFR binding to the target proteins on the surfaces of A431(c), and 293T(d) exosomes. The protein levels are estimated by normalizing the target-associated changes to those of WGA targets.

The content levels of exosome biomarkers can be estimated by normalizing the target-associated changes to those of specific biomarkers abundant in and characteristic of exosomes,^13^ thus allowing us to quantify the biomarker profiles by monitoring the exosome response to different antibodies. To demonstrate this, we flowed the A431 and 293T EV solution onto the anti-CD63-modified sensor surface and counted single-binding events to achieve the image intensity histograms (Fig. 4a and b). After Gaussian fitting, the exosomes have mean diameters of ∼105.7 nm for A431 and ∼108.5 nm for 293T, agreeing well with the NTA analysis results (Fig. S3†). Then, 10 μg mL^−1^ anti-CD81 antibody, 10 μg mL^−1^ anti-epidermal growth factor receptor (anti-EGFR) antibody, and 100 μg mL^−1^ WGA solutions were flowed in a serial onto the A431 and 293T exosomes, respectively. Buffers were flowed in after each protein solution to measure the dissociation. The PSM image intensity response was recorded in real time to produce the binding kinetics curves (Fig. 4c and d). The *k*_on_, *k*_off_, and *K*_D_ values can be determined by fitting the curves with the first-order binding kinetics model as shown in Table 1. After normalizing the CD81 and EGFR-associated changes to those of *N*-acetylglucosamine and sialic acid groups on the exosome surfaces, we can find that the CD81 content level is similar for both A431 and 293T exosomes, while the EGFR content level is much higher on A431 exosome surfaces than 293T exosome surfaces. This is expected because the CD81 is a membrane protein abundant in all exosomes,^53^ and the EGFR was overexpressed on A431 membranes,^27,54^ demonstrating that the PSM can measure content levels and binding kinetics of multiple biomarkers on exosome surfaces for biomarker profiling.

**Table 1:** Association rate constants (*k*_on_), dissociation rate constants (*k*_off_), and equilibrium constants (*K*_D_) for different proteins binding to targets in the A431 and 293T exosomes

	*k*_on_ (M^−1^ s^−1^)	*k*_off_ (s^−1^)	*K*_D_ (nM)
Anti-CD81 to CD81 on A431 exosomes	5.6 × 10^5^	9.7 × 10^−4^	1.7
Anti-CD81 to CD81 on 293T exosomes	6.9 × 10^4^	3.0 × 10^−3^	43.5
Anti-EGFR to EGFR on A431 exosomes	4.7 × 10^5^	6.5 × 10^−4^	1.4
Anti-EGFR to EGFR on 293T exosomes	1.2 × 10^6^	9.0 × 10^−4^	0.8
WGA to *N*-acetylglucosamine and sialic acid groups on A431 exosomes	1.2 × 10^4^	6.9 × 10^−4^	57.5
WGA to *N*-acetylglucosamine and sialic acid groups on 293T exosomes	2.1 × 10^4^	5.3 × 10^−4^	25.2

## Conclusions

We have demonstrated that the PSM enables the exosome size and biomarker analysis with a single instrument, while these measurements need to be respectively finished in NTA and western blot equipment for traditional approach. This ability can simplify the operation, suppress the measurement error resulting from batch-to-batch heterogeneity, and permit selecting the exosome for analysis from the EV solution, which contains many kinds of vesicles with overlapped size distribution. The PSM can also provide a millimetre-scale field of view, similar to a prism-coupled SPR sensor and ∼40 times larger than SPR microscopy, which can capture sufficient exosomes for statistical size analysis. Furthermore, the PSM provides similar SNR to SPR microscopy for single exosome detection and eliminates the irregular particle scattering patterns, which are usually shown in SPR microscopy images, thus allowing the simple automatic processing with open-source ImageJ software. Besides, the binding kinetics and content levels of biomarkers of exosomes can be determined by monitoring the PSM image intensity variations during serially flowing the different antibodies onto the exosomes, providing a label-free and rapid solution for clinical multiplexed biomarker analysis and exploring the exosome surface protein-binding properties. Finally, the PSM in this study can be easily constructed by adding commercially available components to the classical prism-based SPR systems, which have been widely used in commercial SPR products and home-built setups. Thus, we believe that this work will provide an economical and powerful tool for clinical exosome analysis and exploration of fundamental issues such as exosome membrane protein properties.

## Methods

### Materials

Polystyrene nanoparticles were purchased from Bangs Laboratories (Fishers, Indiana, US). The No. 1 cover glasses (22 × 22 mm, catalog no. 48366-067) and 150 mm culture dishes (catalog no. 734-2322) were purchased from VWR (Radnor, PA, US). The gold pellet evaporation materials (catalog no. EVMAU50SHOT) were purchased from Kurt J Lesker (Jefferson Hills, PA, US). Gold-coated glass slides were fabricated by coating a cover glass with 1 nm of Cr followed by 47 nm of gold *via* thermal evaporation (PVD75 E-beam/Thermal Evaporator, Kurt J. Lesker Company). Before coating, the gold surface was rinsed with ethanol and deionized water twice. The microfluidic ball valves were purchased from Cole-Parmer (Vernon Hills, IL, US). Stainless steel dispensing needles (catalog no. KDS2112P) were purchased from Weller (Besigheim, Germany). Dithiol alkane aromatic PEG6–COOH (catalog no. SP35140) was purchased from Nanoscience Instruments (Phoenix, AZ, US). Then, 20 mL syringe (catalog no. 302830) was purchased from BD (Franklin Lakes, NJ, US). Microbore tubes (catalog no. AAD04103) were purchased from Tygon Tubing (Courbevoie, France). Ultracentrifuge bottles (catalog no. 355622) were purchased from Beckman Coulter (Pasadena, CA, US). Dulbecco's modified Eagle's medium (DMEM, cat. no. 20-2002) was purchased from ATCC (Manassas, VA, US). Fetal bovine serum (FBS, cat. no. 10437036), trypsin–EDTA (0.05%, cat. no. 25300120), 1-ethyl-3-(3-dimethylaminopropyl)carbodiimide hydrochloride (EDC, catalog no. 22980), and sulfo-NHS (*N*-hydroxysulfosuccinimide, catalog no. 24510) were purchased from Thermo Scientific (Waltham, MA, US). The FBS was inactivated by heating to 56 °C for 30 minutes. Phosphate-buffered saline (PBS, catalog no. 21-040-CV) and 25 cm^2^ flask (cat. no. 3289) were purchased from Corning (Corning, NY, US). The penicillin–streptomycin mixture (cat. no. DE17-602F) was purchased from Lonza (Basel, Switzerland). Wheat germ agglutinin (WGA, cat. no. L0636) was purchased from Sigma-Aldrich (St. Louis, MO, US). Anti-EGFR (cat. no. 05-101) monoclonal antibody and 0.1% gelatin solution (cat. no. ES-006-B) were purchased from the EMD Millipore (Burlington, MA, US). Anti-CD63 (cat. no. 556019) and anti-CD81 (cat. no. 551112) monoclonal antibodies were purchased from the BD Biosciences (Franklin Lakes, NJ, US). The storage buffer for the proteins was removed with Zeba spin desalting columns (cat. no. 89882, ThermoFisher) before the experiments. Deionized water with a resistivity of 18.2 MΩ cm^−1^ was filtered with 0.22 μm filters (Millex-GS, catalog no. SLGSM33SS) from Sigma-Aldrich (St. Louis, MO, US) and used in all experiments.

### Cell culture and EV isolation

The A431, HeLa, and 293T cells were purchased from ATCC. All the cells were grown in the culture media prepared by mixing DMEM with 10% FBS and 1% penicillin–streptomycin mixture. The culture media has been depleted of exosomes by ultracentrifugation at 120 000*g* for 6 hours. We collected EVs released by HeLa cells with the following steps. First, the HeLa cells were cultured in a 25 cm^2^ flask at 37 °C with 5% CO_2_ and 70% relative humidity. Second, the HeLa cells were passaged with 0.05% trypsin–EDTA when they were approximately 80% confluent in the flask and seeded into the 150 mm culture dishes. Third, the HeLa cells were passaged with 0.05% trypsin–EDTA when they were approximately 80% confluent in the culture dishes and seeded into another ten of 150 mm culture dishes. Fourth, after culturing for 3 days, the supernatant was collected from the ten of 150 mm culture dishes. Fifth, dead cells were depleted from the supernatant by centrifugation at 125*g* for 5 minutes. Sixth, cell debris were depleted from the supernatant by centrifugation at 4000*g* for 30 minutes. Seventh, microvesicles were depleted from the supernatant by centrifugation at 10 000*g* for 30 minutes. Eighth, EVs were collected by ultracentrifugation at 120 000*g* for 4 hours 15 minutes. Ninth, EVs were resuspended by washing the ultracentrifugation bottle wall by ten times and immersion over night with PBS. Tenth, EVs were collected from the solution by ultracentrifugation at 120 000*g* for 4 hours 15 minutes and resuspended by washing the ultracentrifugation bottle wall by ten times and immersion over night with 1 mL PBS. Eleventh, the EV solution was aliquoted and stored at −80 °C. The EVs released by A431 and 293T cells were collected with the same steps.

For negative-stain TEM analysis, 5 μL of EV solution was placed on a formvar/carbon-coated grid and allowed to settle for 1 min. Then, the sample was negative-stained with four successive drops of 1.5% uranyl acetate and washed with distilled water. After air-drying, the grids were imaged with a Philips CM 12 transmission electron microscope (TEM).

### NTA

EV concentration and size distribution were determined using a NanoSight NS300 (Malvern Panalytical, Malvern, UK) equipped with a green laser and a high-sensitivity sCMOS camera following the manuals. Each sample was diluted 10 to 1000-fold in PBS buffer to achieve ∼50 particles in one frame for optimal counting and then introduced into the instrument using a micropump with a 1 mL syringe.

### Experimental setup

The PSM was constructed on a classical prism-coupled SPR system. Light from a laser diode with a center wavelength of 660 nm (OBIS LX 660 nm 75 mW Laser System, Fiber Pigtail, Coherent, Santa Clara, CA, US) was conditioned by three lenses configured in a 4-f arrangement and then excite the SPR on the gold-coated glass slide placed on a prism (cat. no. 49431, Edmund Optics, Barrington, NJ, US). The scattered surface plasmonic waves were collected by a 10× dry objective (NA 0.28, MOTIC, Xiamen, China) and imaged by a USB 3.0 CMOS camera (MC124MG-SY, XIMEA, Münster, Germany). A flow cell is designed for sample delivery. A rectangle double-sided tape (9628B, 3 M, Saint Paul, MN, US) spacer is bound between a No. 1 cover glass with two 1 mm drilled holes (as inlet and outlet) and a gold-coated glass slide to form a flow cell. The cell height is set to be ∼50 μm by controlling the thickness of double-sided tape. Two PDMS blocks with holes created by disposable biopsy punch 0.75 mm with plunger (catalog no. 18271P, Robbins Instruments, Houston, TX, US) were attached to the cover glass after plasma cleaning and incubated at 90 °C for 1 hour to fix the tubes. A push–pull two-way flow mode was configured for sample delivery. The sample was placed in a tube higher than the flow cell, thus allowing the gravity to push the samples thorough the sensor surface. At the same time, a syringe pump (TSD01-01, Lead Fluid, Baoding, China) connected to the outlet was employed to pull the samples to eliminate the flow rate gradient, thus decreasing the pressure on the flow cell wall. This configuration does not require strong seal, thus allowing easy construction of the flow cell. More detailed schematic representation of the optics can be found in Fig. S1.†

### Surface functionalization

Gold-coated glass slides were incubated in 1 mM dithiol alkane aromatic PEG6–COOH in PBS buffer over night to be modified with carboxyl groups. Then, the surface was incubated in 0.05 M NHS/0.2 M EDC for 30 min to activate the carboxyl groups. After rinsing with PBS, 33 nM anti-CD63 was applied to the surface and incubated for 1 hour to allow immobilization *via* EDC/NHS coupling reaction. Lastly, the surface was incubated in 1 mg mL^−1^ BSA for 10 min to block non-specific binding sites.

## Data availability

All relevant data have been included in the figures and ESI.†

## Author contributions

P. Z. performed the experiments and data analysis. J. J. contributed to the EV isolation. X. Z., J. K. and R. W. contributed to the cell culture. X. Z. and Z. W. prepared gold-coated glass slides. S. W. conceived and supervised the project. P. Z. and S. W. wrote the manuscript.

## Conflicts of interest

A US provisional patent application (63/352929) has been filed by the Arizona Board of Regents on behalf of Arizona State University for methods and related aspects for analyzing exosomes based on an early draft of this article. Inventors are S. W. and P. Z.

## Supplementary Material

SC-013-D2SC05191E-s001

SC-013-D2SC05191E-s002
