# Supplementary material for: Label-free imaging and biomarker analysis of exosomes with plasmonic scattering microscopy
Source: Chem Sci. 2022 Oct 12;13(43):12760–8. doi: 10.1039/d2sc05191e (PMC9645376; doi:10.1039/d2sc05191e)
Supplement: SC-013-D2SC05191E-s002 [file SC-013-D2SC05191E-s002.pdf]

Electronic Supplementary Material (ESI) for Chemical Science.
This journal is © The Royal Society of Chemistry 2022

Label-free imaging and biomarker analysis of exosomes with plasmonic scattering microscopy

Pengfei Zhang,[a,b] Jiapei Jiang,[a,c] Xinyu Zhou,[a,c] Jayeeta Kolay,[a] Rui Wang,[a,d] Zijian Wan,[a,e] and Shaopeng Wang *[a,c]

[a.] Biodesign Center for Bioelectronics and Biosensors, Arizona State University, Tempe, Arizona 85287, USA.

[b.] Beijing National Laboratory for Molecular Sciences, Key Laboratory of Analytical Chemistry for Living Biosystems, Institute of Chemistry, Chinese Academy of Sciences, Beijing, 100190, China

[c.] School of Biological and Health Systems Engineering, Arizona State University, Tempe, Arizona 85287, USA.

[d.] State Key Laboratory of Bioelectronics, School of Biological Science and Medical Engineering, Southeast University, 2 Sipailou, Nanjing 210096, China

[e.] School of Electrical, Energy and Computer Engineering, Arizona State University, Tempe, Arizona 85287, USA.

*E-mail: Shaopeng.Wang@asu.edu

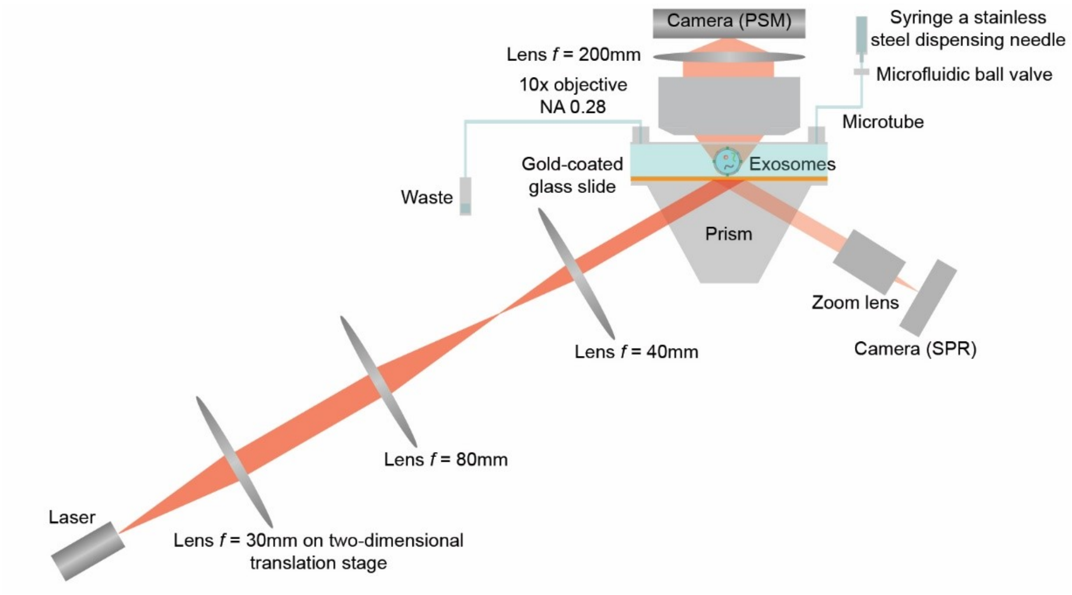

Fig. S1 Optical setup for plasmonic scattering microscopy. Light from a 660 nm laser diode (OBIS LX 660 nm 75 mW Laser System, Fiber Pigtail, Coherent, Santa Clara, CA, US) was conditioned by three lenses configured in a 4-f arrangement. Three lenses have the focus length of 30 mm, 80 mm, and 40 mm, respectively. The distance between laser and first lens, between first and second lens, between second and third lens, and between the third lens and prism surface are set to be 30 mm, 110 mm, 120 mm, and 40 mm to construct the 4-f arrangement. The 4-f arrangement allows scanning of the incident angles without changing the illumination area. The illumination area has an ellipse shape with a size of ~1.8 mm × 1.2 mm due to the oblique illumination configuration. The scattering of surface plasmonic waves by analytes was collected by a 10x objective (NA 0.28, MOTIC, Xiamen, China) on the top of a gold-coated glass slide, and then imaged by the camera (MC124MG-SY, XIMEA, Münster, Germany) to form the PSM image. The reflection light was collected by a zoom lens (VZM™ 1000 Zoom Imaging Lens, Edmund Optics, Barrington, NJ, US) with a 4x magnification, and then imaged by the camera (MC023MG-SY, XIMEA, Münster, Germany) to form the SPR image. The sample was stored in a 20 mL syringe, and flowed onto the surface with a stainless steel dispensing needle and microbore tubes. A microfluidic ball valve was employed to control the sample flow.

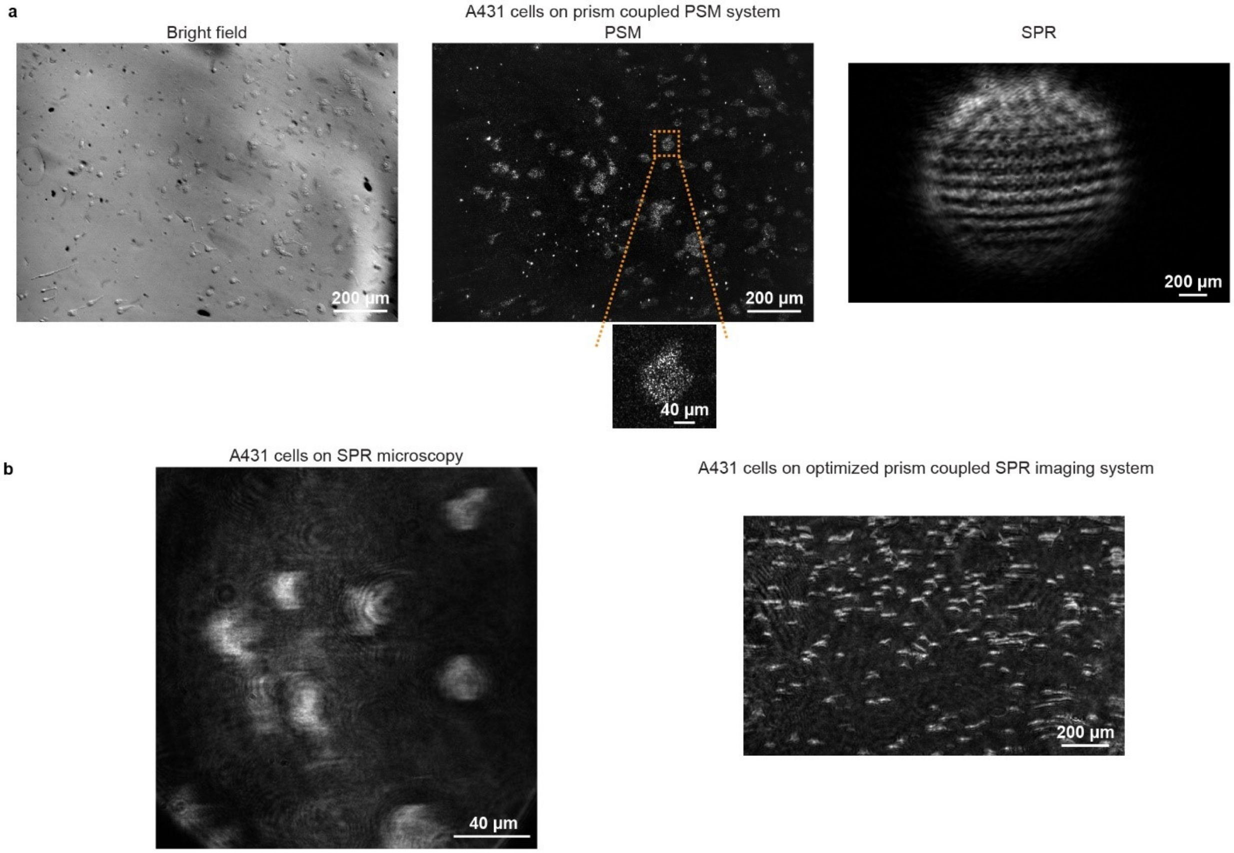

Fig. S2 a, Bright field, PSM and SPR images of the A431 cells in the prism coupled PSM system. Because the laser is used in this system to achieve high incident intensity for PSM, the interference effect covers the SPR images, making it only suitable for ensemble SPR measurement. **b**, SPR images of the A431 cells in another optimized prism coupled SPR imaging system. It can be seen that the PSM provides a high spatial resolution to image the cell adhesion sites, which has been revealed by total internal reflection fluorescence microscopy (Journal of cell science 2010, 123(21), 3621-3628). These results show that the PSM can provide higher spatial resolution than traditional SPR imaging systems.

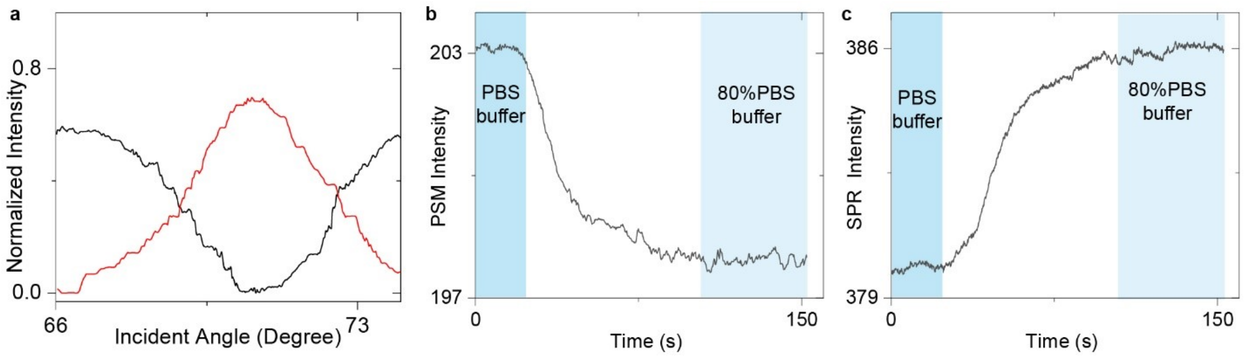

Fig. S3 a, Experimental SPR curve and PSM scattering curve. The intensity is achieved by averaging the intensities of all pixels in the raw image. **b**, PSM intensity response during changing the PBS buffer to 80% PBS buffer in water, where the PSM intensity variation is ~5.18 grayscales. The standard deviation σ of PSM intensity measuring PBS buffer is ~0.063 grayscale. The refractive index variations between PBS buffer and 80% PBS buffer can be estimated to be (46 mDeg)/(130 Deg/RIU) ~ 3.54×10^{-4} RIU, where 46 mDeg is the ensemble SPR intensity difference between 100% and 80% PBS buffer (Nat Methods 2020, 17, 1010–1017), 130 Deg/RIU is the ensemble SPR sensitivity factor, and RIU represents refractive index unit. Then, the sensitivity factor (SF) of PSM channel can be estimated by (5.18 grayscales)/(3.54×10^{-4} RIU) ~ 1.46×10^{4} grayscales/RIU. Finally, the refractive index resolution of PSM for ensemble measurements can be determined to be σ / SF = (0.063 grayscale)/(1.46×10^{4} grayscales/RIU) ~ 4.3×10^{-6} RIU, which is comparable to most ensemble SPR sensors (Chemical Reviews 2008, 108 (2), 462-493). **c**, SPR intensity response during changing the PBS buffer to 80% PBS buffer in water, where the PSM intensity variation is ~6.02 grayscales. The standard deviation σ of SPR intensity measuring PBS buffer is ~0.11 grayscale. Using the same protocol as Fig. S3b, the sensitivity factor SF of SPR channel can be estimated by ~ 1.70×10^{4} grayscales/RIU, and the refractive index resolution of SPR channel can be estimated to be ~ 6.4×10^{-6} RIU, which is comparable to most ensemble SPR sensors (Chemical Reviews 2008, 108 (2), 462-493).

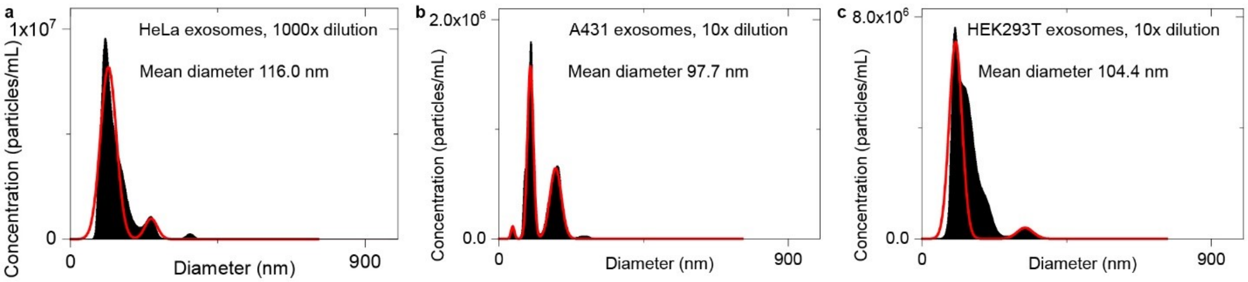

Fig. S4 Size distributions of extracellular vesicles from different cells measured by nanoparticle tracking analysis instrument (NanoSight NS300, Malvern Panalytical, Malvern, UK). The solid lines are Gaussian fittings. The EV sample was diluted before measurement. The dilution factor for achieving ~50 vesicles in one frame and the mean diameter of the vesicles are marked in the figures.

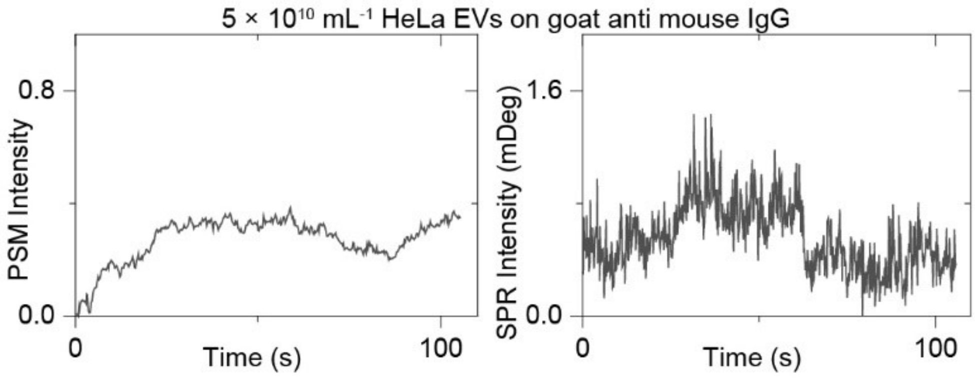

Fig. S5 Ensemble PSM and SPR measurement of 5×10^{10} mL^{-1} HeLa EVs binding to the goat anti mouse IgG antibody. Compared with Fig. 2, we can see that the nonspecific binding is very weak.

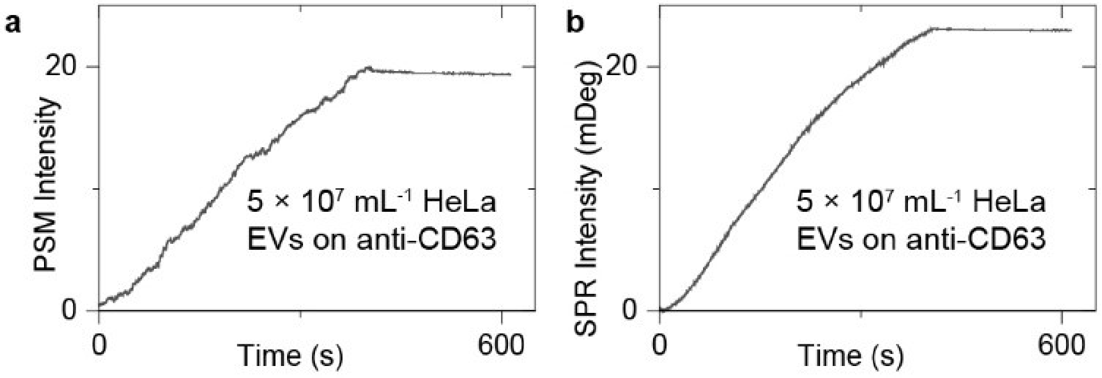

Fig. S6 Ensemble PSM and SPR measurement of 5 × 10^7 mL^{-1} HeLa EVs binding to the anti-CD63 antibody. The curve is hard to fit because no obvious dissociation was observed within the measurement period.

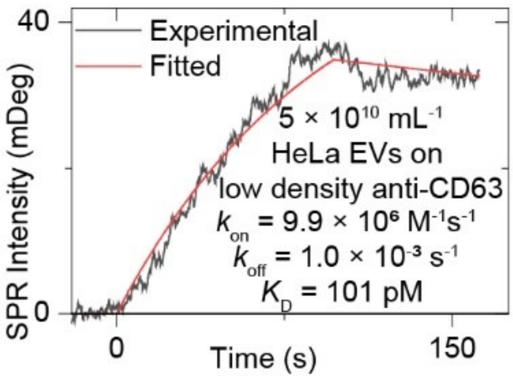

Fig. S7 Ensemble measurement of 5×10^{10} mL^{-1} HeLa EVs binding to the low-density anti-CD63 antibody. The low-density anti-CD63 modified sensor surface by incubating the gold surface with the solution mixing 20 nM BSA with 20 nM anti-CD63antibodies.

Note S1. Effective diameter correction

The surface plasmon field decreases exponentially from the surface (*z*-direction) into the solution. In other words, the scattering of the evanescent field by a finite-size object depends on the distance (*z*) from the surface. The effective scattering diameter D_{eff} and volume V_{eff} of the analyte can be given by

$$\frac{4}{3}\pi\left(\frac{D_{eff}}{2}\right)^3 = V_{eff} = \int_0^{2R} \pi\left(R^2 - (R - z)^2\right)e^{-\frac{z}{l}}dz = \int_0^{2R} \pi\left(Dz - z^2\right)e^{-\frac{z}{l}}dz, \qquad (S1)$$

where *z* is the distance from the gold surface, *R* is the radius of the analyte, *D* is diameter of analyte, and $l = 100$ nm is the decay length of the evanescent field (Fig. S4; PNAS, 2010, 107(37): 16028-16032). The effective diameters for polystyrene nanoparticles with hydrodynamic diameters of 93.7 nm, 143.6 nm, and 190.7 nm are 80.7 nm, 115.0 nm, and 143.0 nm, respectively.

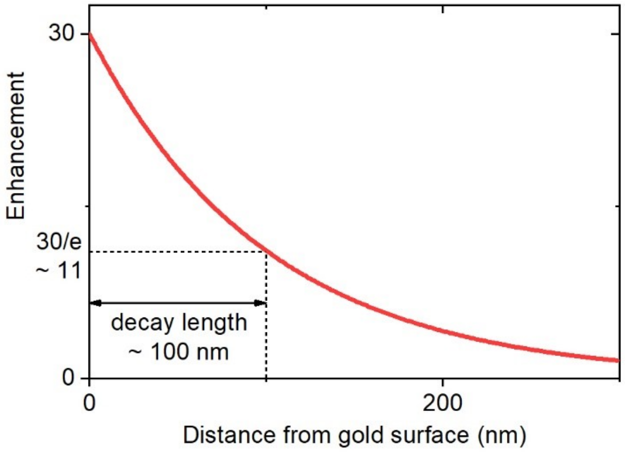

Fig. S8 Surface plasmon field intensity distribution calculated with transfer matrix approach (Journal of Sensors, 2015, 2015, 945908).

Note S2. Signal-to-noise ratio analysis

To estimate the theoretical singal-to-noise ratio (SNR) limit for the PSM system, the total Rayleigh scattering intensity I_{total} of one small object can be estimated by

$$I_{total} = \frac{\frac{2\pi^5}{3} \times \frac{d^6}{\left(\frac{\lambda}{n_m}\right)^4} \times \left(\frac{\left(\frac{n_s}{n_m}\right)^2 - 1}{\left(\frac{n_s}{n_m}\right)^2 + 2}\right)^2}{A} \times P \times A \times t, \qquad (S2)$$

where n_s and n_m are the refractive indices of analyte and medium, λ is the incident wavelength, d is the analyte diameter, P is the incident light intensity, t is the average period. For the polystyrene nanoparticles measured by PSM here, $P = 4$ W cm^{-2}, $n_s = 1.58$, $n_m = 1.33$, $\lambda = 660$ nm, and $t = 0.1$ s. Considering the single photon energy of ~1.2398/(0.66 μm) eV and the 30 x intensity enhancement of surface plasmon field, the total scattering intensity of one object in the PSM system can be expressed as

$$I_{total} = 2 \times 10^{-5} \times (d(nm))^6 photons. \qquad (S3)$$

The objective collects the scattering photons in perpendicular to the propagation direction of surface plasmon wave, and the collection efficiency can be calculated with the equation in spherical coordinate system of

$$\frac{I_{collection}}{I_{total}} = \frac{\int_{\theta_1}^{\theta_2}\int_{\varphi_1}^{\varphi_2} \frac{(1 + cos^2\theta)}{R^2} R^2 sin\theta d\varphi d\theta}{\int_0^{\pi}\int_0^{2\pi} \frac{(1 + cos^2\theta)}{R^2} R^2 sin\theta d\varphi d\theta} = \frac{\varphi_2 - \varphi_1}{2\pi} \times \frac{\int_{\theta_1}^{\theta_2} (sin\theta + sin\theta cos^2\theta) d\theta}{\int_0^{\pi} (sin\theta + sin\theta cos^2\theta) d\theta}, \qquad (S4)$$

where θ and φ are the polar angle and azimuthal angle, respectively. The objective collection angle for the PSM can be calculated by

$$\vartheta = arcsin\left(\frac{NA}{n_m = 1.33}\right), \qquad (S5)$$

where NA is the objective numerical aperture, and $n_m = 1.33$ is used to correct the effect of water refraction on the scattering light collection. For the objective with NA of 0.28, the collection efficiency is calculated to be ~1.1 %. For one polystyrene nanoparticle with effective diameter of 80.7 nm, the objective can collect ~60766 scattering photons, which can be converted to 32814 electrons after considering the camera quantum efficiency of 54% at incident wavelength of 660 nm. The camera sensitivity is ~2.4 electrons/grayscale. Thus, one polystyrene nanoparticle with real diameter of 93.7 nm and effective diameter of 80.7 nm can produce the total intensity of ~13672 grayscales in the image, agreeing with the experimental results of 13040 grayscales. The standard deviation of intensities of images recorded in the absence of nanoparticles is ~90 grayscales. Thus, the SNR of our system measuring 93.7 nm polystyrene nanoparticle is determined to be ~145.

Note S3. Refractive index correction

The extracellular vesicles, including the exosomes, own the refractive index of 1.39 (Journal of Extracellular Vesicles 2014, 3, 25361), and polystyrene nanoparticles own the refractive index of 1.58 (refractiveindex.info). Based on the equation S2, the extracellular vesicles will have ~16 times smaller intensity than the polystyrene nanoparticles with the same diameter.

Note S4. Exosome concentration estimation

On the basis of Fick's law of diffusion, one could expect the binding frequency of analytes decreased with time, as described in the classical Cottrell equation

$$f(t) = \frac{CAD^{0.5}}{\pi^{0.5}} t^{-0.5}, \#(S6)$$

where *f(t)* is the binding frequency as a function of time, *A* is the area of the observation region, *D* and *C* are the diffusion coefficient and the concentration of nanoparticles, respectively (Anal. Chem. 2016, 88, 2380–2385). After integration, one can estimate the number of total binding events *F(t)* to be

$$F(t) = \frac{2CAD^{0.5}}{\pi^{0.5}} t^{0.5}. \#(S7)$$

The analyte diffusion coefficient can be estimated with Stokes–Einstein equation

$$D = \frac{k_B T}{6\pi\eta r}, \#(S8)$$

where k_B is the Bolzmann constant, *T* is temperature, η is the viscosity of the liquid, *r* is the hydrodynamic radius. For exosomes with diameter of ~100 nm, the diffusion coefficient can be estimated to be 4.9×10^{-12} m^2/s.

Within the period of 30 s, the exosome concentration can be estimated to be ~1×10^7 mL^{-1},and ~8×10^9 mL^{-1} for 201, and 1.61×10^5 binding events, respectively.
